# Aminaphtone in the control of Schamberg's disease

**DOI:** 10.1186/1477-9560-7-8

**Published:** 2009-06-11

**Authors:** José Maria Pereira de Godoy, Fernando Batigália

**Affiliations:** 1 Angiology and Vascular Surgery Service, São José do Rio Preto Medicine School, SP (FAMERP), Brazil; 2 São José do Rio Preto Medicine School, SP (FAMERP), Brazil

## Abstract

The aim of this case report is to describe control of Schamberg's disease using aminaphtone. We report on the case of a 28-year-old patient who presented with multiple purpuric lesions of the lower extremities which had appeared spontaneously. A biopsy of the skin was performed that showed a perivascular T-cell lymphocytic infiltrate centered on the small superficial blood vessels of the skin and so a diagnosis of Schamberg's disease was reached. The patient was prescribed corticoids and the lesions disappeared however on suspension of the medication the lesions re-emerged within three to seven days. This treatment was unsuccessfully continued for more than one year. Thus another therapeutic option was attempted: 75 mg of aminaphtone was prescribed twice daily for one month and the purpuric lesions disappeared within about one week. One year after suspending the medication no relapse of the purpura was observed.

## Introduction

Since progressive pigmented purpuric dermatitis, also known as Schamberg's disease, was first described in 1901, several articles have appeared that have dealt largely with clinical and histologic features of the illness[1]. The lesions may occur in any location but most often affect the lower extremities. The skin lesions are nonpalpable macules, which are typically asymptomatic, may persist for months to years; normally only the aesthetic appearance is of concern to patients [2].

Histologic examinations show a lymphocytic vasculitis involving the blood vessels of the upper dermis with endothelial swelling and extravasated red blood cells[3].

Several therapies have been used including topical and systemic corticosteroids, vitamin C and topical and systemic anti-inflammatory agents[4]. As aminaphtone has been used to treat patients with changes in capillary permeability and fragility[5,6], it may be useful in the control of Schamberg's disease. The aim of this case report is to describe the treatment of Schamberg's disease using aminaphtone.

## Case report

**Figure 1:**
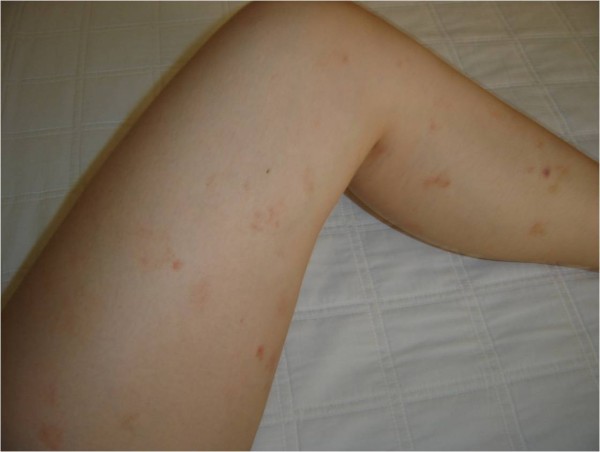
**Purpuric lesions on the lower extremities of the 28-year-old female patient**.

We report on the case of a 28-year-old patient who, at the age of 24, presented with multiple purpuric lesions of the lower extremities which appeared spontaneously (Figure 1). Biochemical examinations, a complete blood test and coagulogram all gave normal results. A biopsy of the skin was then performed that showed a perivascular T-cell lymphocytic infiltrate centered on the small superficial blood vessels of the skin and so a diagnosis of Schamberg's disease was reached. The patient was prescribed corticoids and the lesions disappeared however on suspension of the medication the lesions re-emerged within three to seven days. This treatment was unsuccessfully continued for more than one year. Thus another therapeutic option was attempted: 75 mg of aminaphtone was prescribed twice daily for one month and the purpuric lesions disappeared within about one week. One year after suspending the medication no relapse of the purpura was observed. This study was approved by the Ethics Committee of Medical School of São Jose do Rio Preto, Brazil (FAMERP).

## Discussion

The current report describes the control of purpuric lesions using aminaphtone for a young female patient with Schamberg's disease. The etiology of this disease remains relatively unknown and there is no consensus to which medication is best for its control, perhaps due to the scarcity of published case reports.

The use of aminaphtone in this disease aims at improving capillary fragility and, in this case it successfully controlled the purpuric lesions. The use of other medications, such as pentoxifylline, corticoid and vitamin C, has also been described. This new option seems to be attractive due to the few side effects reported for the medication and the long periods without the reappearance of the purpuric lesions. However further studies are necessary to evaluate its efficacy over the long term.

## Consent

The authors certifies that his institution has approved the protocol (n°0059.0.000.140-09Brazil)for any investigation involving humans or animals and that all experimentation was conducted in conformity with ethical and humane principles of research of University Medical School of São Jose do Rio Preto-Brazil-FAMERP. The consent term was written and signed.

## Competing interests

The authors declare that they have no competing interests.

## Authors' contributions

All authors confirm the participation in study in all of the phases and agree with the content of the manuscript.
